# Single-cell chromatin landscapes of mouse skin development

**DOI:** 10.1038/s41597-022-01839-9

**Published:** 2022-12-02

**Authors:** Fang Li, Tiantian Xu, Jiale Li, Xuxu Hao, Wei Ge, Xin Wang

**Affiliations:** 1 Key Laboratory of Animal Genetics, Breeding and Reproduction of Shaanxi Province, College of Animal Science and Technology, Northwest A&F University, Yangling, Shaanxi 712100 China; 2 College of Life Sciences, Qingdao Agricultural University, Qingdao, 266109 China

**Keywords:** Epithelial-mesenchymal transition, Organogenesis

## Abstract

The coat of mammals is produced by hair follicles, and hair follicle is an important and complex accessory organ of skin. As a complex physiological regulation process, hair follicle morphogenesis is regulated by a series of signal pathway factors, involves the interaction of multiple cell types and begins in the early embryonic stage. However, its transcriptional regulatory mechanism is unclear. We have therefore utilized single-cell ATAC sequencing to obtain the chromatin accessibility landscapes of 6,928, 6,961 and 7,374 high-quality cells from the dorsal skins of E13.5, E16.5 and P0 mice (*Mus musculus*), respectively. Based on marker gene activity clustering, we defined 6, 8 and 5 distinct cell types in E13.5, E16.5 and P0 stages, respectively. Furtherly, we integrated the fibroblasts and keratinocytes clusters, performed further analysis and re-clustered. The single cell map of the chromatin open area was drawn from each cell type and the mechanism of cell transcription regulation was explored. Collectively, our data provide a reference for deeply exploring the epigenetic regulation mechanism of mouse hair follicles development.

## Abstract (summary)

**Table:** 

Measurement(s)	Mouse Skin
Technology Type(s)	scATAC-seq (Microfluidics)
Sample Characteristic - Location	China

## Background & Summary

Hair follicle, as one of the skin appendages, is the most tractable model to study appendage development^1^. Hair follicle development involves complex interactions between the epidermis and underlying mesenchyme and is produced from a series of specific sites in the ectoderm and the underlying mesoderm^2^. During hair follicle development, the dynamic morphological changes have been extensively explored^3–5^. The hair follicle development of mice has been histologically categorized into three unique stages: induction (E13.5 - E14.5), organogenesis (E15.5–17.5), and cytodifferentiation (E18.5 onwards) in utero^5^, the molecular and cellular events of those morphological stages have been well characterized. In the induction stage, dermal first signal initiates hair follicle development, placode (Pc) and dermal condensates (DC) structures are gradually formed. In the organogenesis stage, keratinocyte proliferation leads to the formation of hair germ, further down-growth progresses to the peg stage. In cytodifferentiation stage, the most-proximally located keratinocytes begin to enwrap the dermal papilla (DP), followed by the bulbous peg stage and distinct strata of epithelial differentiation within hair follicle become morphologically noticeable^5^. Identifying the internal and external signaling mechanisms of hair follicle morphogenesis are the key for understanding the dynamic epithelial-mesenchymal interactions during the complex tissue development^6^. A multicellular organism comprises diverse cell types which is highly specialized to carry out unique functions^7^. The establishment of different cell lineage for development relies on specific spatiotemporal gene expression programs^8^ and gene regulatory networks (GRNs)^9^. Transcription factors bind to enhancers and promoters to regulate target gene expression, ultimately resulting in a cell type-specific transcriptome^10–12^. Single-cell technologies provide new opportunities to study the mechanisms underlying cell identity. Single cell RNA sequencing (scRNA-seq) was recently adopted for deciphering hair follicle heterogeneity across cell sub-populations, distinguishing fine molecular differences between individual cells, and describing the transcription atlas of mouse skin hair follicle^1,13^.

Single-cell ATAC sequencing (scATAC-seq), serving as a read-out of chromatin accessibility^9^, is a powerful tool to interrogate the epigenetic heterogeneity of cells and reveal cell type-specific transcriptional regulatory network^14^. Recent technical advancements in scATAC-seq have made it possible to simultaneously analyze the open chromatin regions of tens of thousands of cells and list the active DNA regulatory elements profile of the chromatin states such as cis- and trans-regulatory elements^15^. These open chromatin regions play important regulatory roles in distinguishing the cell types from complex organisms^7^. scATAC-seq has an essential role in depicting the trajectories of cell differentiation^15,16^, elucidating the transcriptional regulators of developmental lineages^17^, revealing the complex patterns of gene regulatory relationships for maintaining cellular state and developmental processes^18^.

To systematically investigate the cellular complexity of developing embryonic skin and gain the comprehensive insights into the molecular identity of hair follicle progenitors and niche cells, the nuclei from single-cell suspensions of E13.5, E16.5 and P0 mice dorsal skin were obtained using 10x Genomics Chromium^TM^ Controller &Accessory Kit. Then single-cell libraries were constructed and performed 10x Genomics single-cell ATAC sequencing on a droplet-based commercial platform. The raw data were obtained and subsequent data analysis was processed with Signac^19^. The datasets here provided the single-cell epigenomic profiling of hair follicle cells from the skin at different stages of mouse embryonic development. Our work would provide a suitable reference and basis for future single-cell chromatin studies, enriching the spectrum of cellular heterogeneity with hair follicle development and the dynamic morphological changes, serving as a valuable resource to understand how the system changes during hair follicle morphogenesis.

## Materials and Methods

### Ethics statement

All experimental protocols were approved by the Experimental Animal Manage Committee of Northwest A&F University (2011-31101684).

### Isolation of mononuclear cells from mice skins

The dorsal skin tissues were collected from the pregnant mice on E13.5, E16.5 and postnatal day 0 (P0). Initially, the dissected skins were incubated with TrypLETM Express (TE, 1X) (Gibco) at 37 °C for 30 min, then separated the epidermis and dermis under a stereoscope (Motic). Thereafter, the epidermis was digested with TE for 15 min, while the dermis was digested with 2 mg/mL collagenase type II (Sigma, St Louis, MO, USA) for 15 min. Then the cells were centrifuged and resuspended in phosphate-buffered solution (PBS) containing 0.04% bovine serum albumin (BSA). Eventually, the cell suspensions were filtered through a 40-μm mesh and completed the preparation of single-cell suspension.

### Nuclei isolation and scATAC-seq library preparation

The concentration of cell suspension was counted using a hemocytometer (TC20, Bio-Rad, Hercules, CA, USA) immediately, and the cell membrane was destroyed by surfactant, then the nuclear suspension was prepared. The nuclei concentrations were measured and adjusted to the desired capture number. The single nuclear barcoding and library preparation were performed following the 10x Chromium Single Cell ATAC Library & Gel Bead Kit (16 rxns PN-1000110) and sequenced on the Illumina NovaSeq. 6000 (Illumina, San Diego, CA, US) platform. Finally, 8016, 7714 and 7896 single nuclear samples from E13.5, E16.5 and P0 stages were sequenced, respectively.

### Raw data processing

Preliminary sequencing data was transformed into FASTQ format using Cell Ranger ATAC (version 1.2.0, https://cf.10xgenomics.com/releases/cell-atac/cellranger-atac-1.2.0.tar.gz) by 10x Genomics standard sequencing protocol. Then the FASTQ files were aligned to mouse genome reference sequence mm10 (GRCm38.p6) using cell ranger ATAC count. Subsequently, we applied Cell Ranger for preliminary data analysis and generated a file that contained barcoded BAMs, peaks.bed, fragments tsv.gz, per barcode cell calling etc. Eventually, the output files (pre-process data) were used for the downstream visualization analysis.

### Bioinformatic analysis of scATAC-seq data

#### Quality control (QC) filtering

R (version 3.6.1, https://www.r-project.org/) and Signac R packages (version 1.0.0, https://github.com/timoast/signac/)^20,21^ were used to perform downstream analysis. We identified barcodes representing genuine cells mainly by TSS enrichment score and the number of unique fragments. The filter metrics were determined by referencing the Signac official tutorial and previous studies (https://satijalab.org/signac/)^20^. The criterion was as follows: (1) the peak region fragment was >3000 and <10000 unique fragments; (2) enrichment at transcription start sites (TSS) ≥2; (3) pct reads in peaks ≥15; (4) blacklist ratio ≤0.025; (5) nucleosome signal <10 were filtered. And the outliers for those QC metrics were removed.

#### Normalization and Linear dimensional reduction

After QC, the high quality scATAC-seq datasets were obtained, then were normalized by term frequency-inverse document frequency (TF-IDF) and Seurat function “Run TFIDF”. The dimensionality was reduced from the DNA accessibility assay by latent semantic indexing (LSI), while the first LSI component was usually be removed from downstream analysis for capturing sequencing depth rather than biological variation.

#### Non-linear dimension reduction and clustering

After linear dimensional reduction, the cells were embedded in a low-dimensional space, performed graph-based clustering and non-linear dimension reduction for visualization, and applied the UMAP algorithm to visualize and identify cell clusters by Seurat function of “RunUMAP” and “FindClusters”.

#### Generating a counts matrix and cell-type annotation of scATAC-seq clusters

To define the specific highly expressed gene set of each cluster, we generated a count matrix and calculated the genescore value by the Signac function “GeneActivity ()”. The activity of each gene was quantified by evaluating the chromatin accessibility associated with the gene in the scATAC-seq data. A gene activity matrix was generated from the reads mapped to gene body and promoter (upstream 2 kb from the TSS), and calculated the genescore value of each gene. In order to facilitate cluster annotation, the gene activity of TopFeatures was examined and visualized genescore by “DotPlot”. Finally, the “gene activity” of some typical cell type-specific marker genes were visualized for clustering and cell type assignment of scATAC-seq data.

## Data Records

**Fig. 1:**
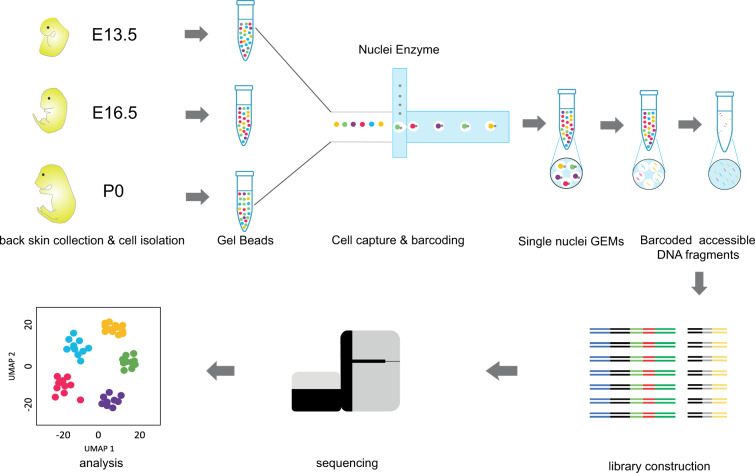
Workflow of mice skin scATAC-seq.

We present chromatin accessibility landscapes of different cell types of mice skins, as a reference to deeply explore the epigenetic regulation mechanism of cell heterogeneity. Our data set on skins consists of chromatin accessibility landscapes for 6928, 6961 and 7374 high-quality cellular (single nuclear), respectively. According to the developmental characteristics of hair follicles at different stages and gene activity of scATAC-seq, we assigned biological identities to 6, 8 and 5 populations based on the gene activity of known marker genes. Figure 1 provides an overview of laboratory and bioinformatical workflow.

All skin scATAC-seq data have been uploaded to the NCBI Gene expression Omnibus (GEO) database with accession number GSE201213^22^. The raw data of the three samples have been deposited in NCBI Sequence Read Archive (SRA) and are accessible through the accession numbers: SRX14951484^23^, SRX14951485^24^ and SRX14951486^25^.

## Technical Validation

All mice dorsal skins used in this study were freshly collected, dissected and digested into single cells (Methods). Each sequencing samples were from three independent individuals mixed in equal proportion according to the same cell count. Increasing biological duplication ensured the reliability of scATAC-seq data.

After sequencing the three libraries on an Illumina NovaSeq. 6000 and processing the raw sequencing data with Cell Ranger ATAC v1.2.0, pre-process data were analyzed with Seurat and Signac. For E13.5, E16.5 and P0 pre-process data, we detected 8,016, 7,714 and 7,896 cells, and obtained a median number of 19,906, 27,044 and 24,006 fragments per cell. All the libraries achieved a high overlapping rate for fragments of 69.3%, 69.9% and 74.9% (>55%) (Table 1). The Q30 index was beyond the QC low-border, indicating that high-quality mapping data were generated for the downstream analysis.

**Table 1:** Overview of the mapping parameters for the 10x Genomics scATAC-seq datasets established in mice skins.

	E13.5	E16.5	P0
Estimated number of cells	8016	7714	7896
Median fragments per cell	19906	27044	24006
Fraction of fragments overlapping any targeted region	69.3%	69.9%	74.9%
Fraction of transposition events in peaks in cell barcodes	50.8%	52.5%	66.1%
Fraction of read pairs with a valid barcode	75.7%	98.4%	97.8%
Q30 bases in Read 1	91.4%	94.3%	91.8%
Q30 bases in Read 2	90.8%	93.8%	91.2%
Q30 bases in Barcode	86.5%	90.3%	86.7%
Q30 bases in Sample Index	90.1%	93.1%	90.9%
Enrichment score of transcription start sites	8.13	8.15	7.31
Fraction of fragments overlapping TSS	31.6%	29.9%	34.0%
Fraction of fragments overlapping called peaks	53.4%	55.5%	68.5%
Fraction of transposition events in peaks in cell barcodes	50.8%	52.5%	66.1%
Fraction of fragments overlapping any targeted region	69.3%	69.9%	74.9%
Fraction of total read pairs mapped confidently to genome (>30 mapq)	66.7%	86.7%	88.6%
Fraction of total read pairs that are unmapped and in cell barcodes	1.0%	1.1%	1.3%
Fraction of total read pairs in mitochondria and in cell barcodes	0.4%	1.2%	0.2%

We further used Signac to filter low-quality data, in which the TSS enrichment and unique fragment from each cell were calculated (Supplementary Fig. 1, available at Figshare^26^). Hence, we computed the nucleosome banding pattern, the total number of fragments in peaks, the fraction of fragments in peaks, ratio reads in ‘blacklist’ sites and transcriptional start site (TSS) enrichment score in each sample and removed the cells with the peak region fragment was >3000 and <10000 unique fragments, enrichment at TSS ≥2, pct reads in peaks ≥15, blacklist ratio ≤0.025, nucleosome signal <10.

After QC, 6,928, 6,961 and 7,374 high-quality nuclei were further analyzed, and the cell clustering was visualized by UMAP. The sample of E13.5 formed 8 indistinct clusters, E16.5 formed 12 separated clusters and P0 formed 13 clusters. Differential gene activity between the clusters was identified. The 20 top differential gene activity per cluster could be found in Supplementary Table 1 (available at Figshare^26^) for E13.5 clusters, Supplementary Table 2 (available at Figshare^26^) for E16.5 clusters, and Supplementary Table 3 (available at Figshare^26^) for P0 clusters, respectively.

Differential gene activity in the pre- and early post-implantation mammalian embryo resulted in the expression of certain parts of the genotypic potential to create a phenotypic form^27^. From the literature, we expected to find several dermal and epidermis cells during hair follicle development, such as epidermal keratinocytes, dermal fibroblasts, neural crest-derived melanocytes, schwann cells, etc^6^.

**Fig. 2:**
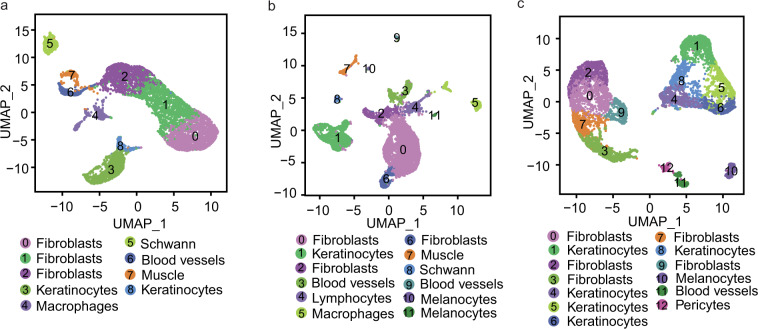
Clustering and UMAP visualization of scATAC-seq data in E13.5 (**a**), E16.5 (**b**) and P0 (**c**) mice skins.

**Fig. 3:**
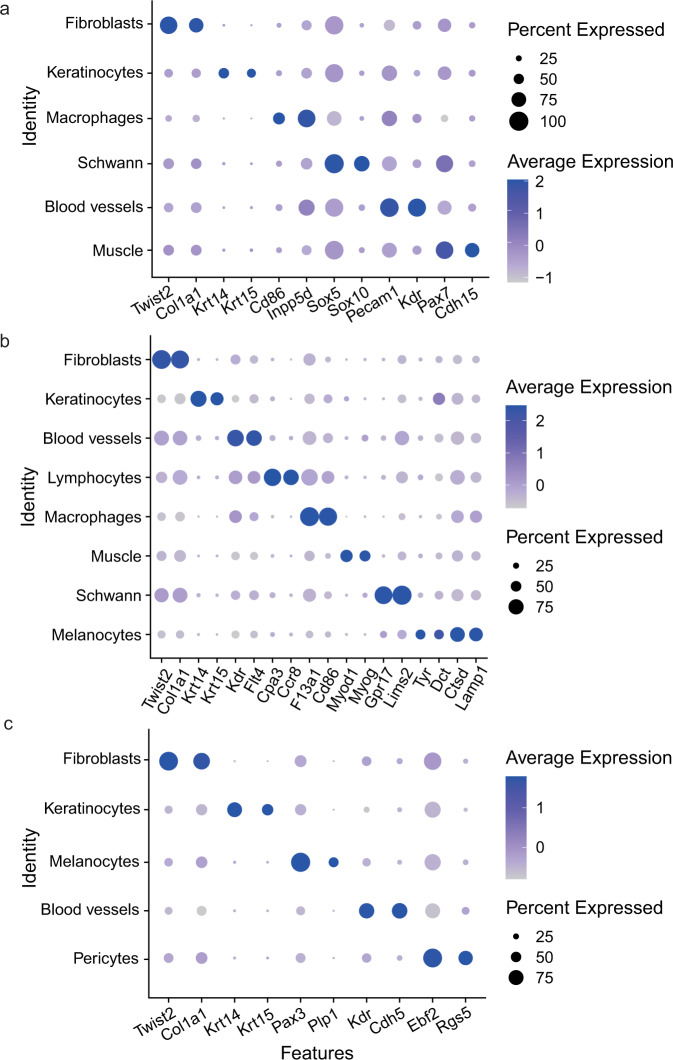
A paired dot plot of scaled expression of selected marker genes for cell type identification in E13.5 (**a**), E16.5 (**b**) and P0 (**c**). The dot size encodes the proportion of cells that express the gene, while the color encodes the scaled average expression level across those cells (dark blue is high).

**Fig. 4:**
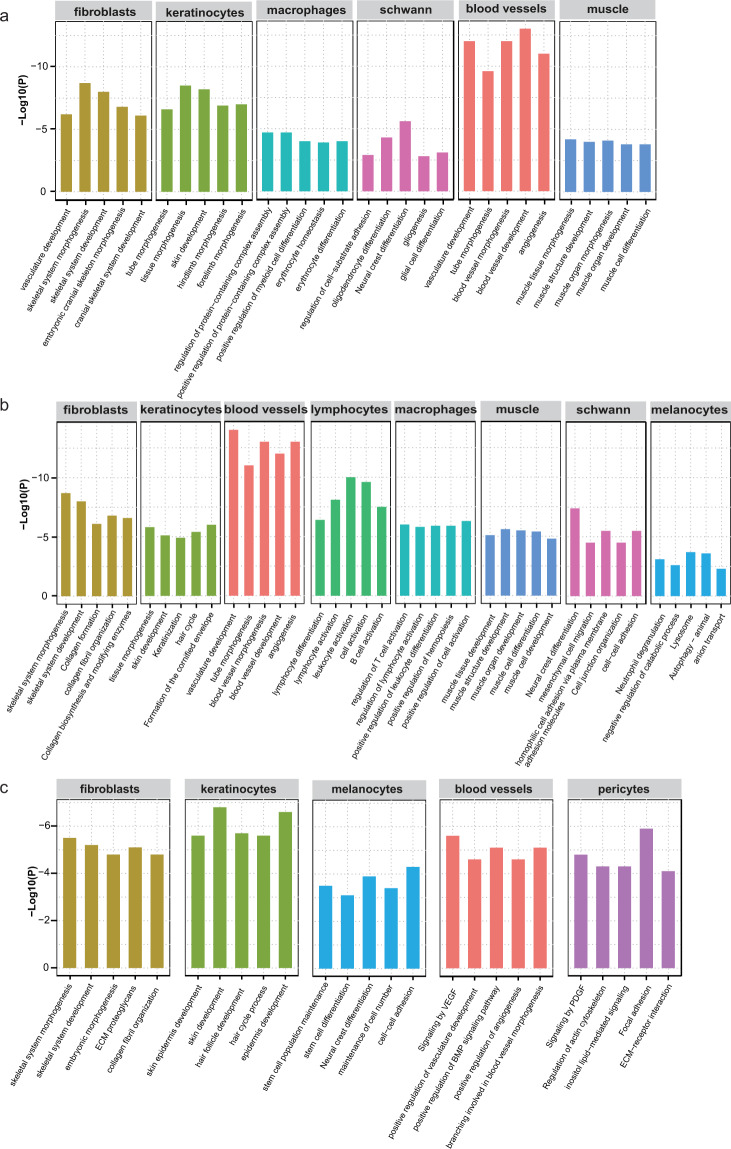
GO enrichment analysis of top20 differentially gene activity in E13.5 (**a**), E16.5 (**b**) and P0 (**c**).

We focused on the “gene activity” of the cluster and the marker gene of different cell-type to validate that the established dataset was indeed represented a hair follicle population. In E13.5, we found that clusters 0, 1 and 2 mainly expressed fibroblast markers of *Twist2*^28^ and *Col1a1*^29^, clusters 3 and 8 expressed keratinocytes markers of *Krt14*^30^ and *Krt15*^31^, cluster 4 expressed macrophages markers of *Cd86*^32^ and *Inpp5d*^33^, cluster 5 expressed schwann markers of *Sox5*^6^ and *Sox10*^34^, cluster 6 expressed blood vessels markers of *Pecam1*^35^ and *Kdr*^36,37^, cluster 7 expressed muscle markers of *Pax7*^30^ and *Cdh15*^38^. In E16.5, we detected that clusters 0, 2 and 6 expressed fibroblasts markers of *Twist2* and *Col1a1*, cluster 1 expressed keratinocytes markers of *Krt14* and *Krt15*, cluster 3 and 9 expressed blood vessels markers of *Kdr* and *Flt4*^36^, cluster 4 expressed lymphocytes markers of *Cpa3*^39^ and *Ccr8*^40^, cluster 5 expressed macrophages markers of *Cd86* and *F13a1*^41^, cluster 7 expressed muscle markers of *Myod1*^42^ and *Myog*^43^, cluster 8 expressed schwann markers of *Gpr17* and *Lims2*^6^, cluster 10 expressed melanocytes markers of *Tyr*^6^ and *Dct*^44^, cluster 11 expressed melanocytes markers of *Ctsd*^6^ and *Lamp1*^45^. In P0, we detected that clusters 0, 2, 3, 7 and 9 mainly expressed fibroblasts markers of *Twist2* and *Col1a1*, clusters 1, 4, 5, 6 and 8 expressed keratinocytes markers of *Krt14* and *Krt15*, cluster 10 expressed melanocyte markers of *Pax3*^6^ and *Plp1*^46^, cluster 11 expressed blood vessels markers of *Kdr* and *Cdh5*^30^, whereas the cells of cluster 12 expressed pericytes markers of *Ebf2*^30^ and *Rgs5*^47^. According to the gene activity of marker genes, the cells were classified into 6, 8 and 5 populations, respectively. The 6 populations included fibroblasts, keratinocytes, macrophages, schwann, blood vessels and muscle (Fig. 2a); the 8 populations were fibroblasts, keratinocytes, blood vessels, lymphocytes, macrophages, muscle, schwann and melanocytes (Fig. 2b); and the 5 populations were fibroblasts, keratinocytes, melanocytes, blood vessels and pericytes (Fig. 2c). The specific markers of these different cell types were shown in Fig. 3. GO term analysis was performed on the identified top 20 differentially gene activity (Fig. 4). The result showed that fibroblasts enriched in the signaling pathways including skeletal system development and embryonic morphogenesis; keratinocytes in the signaling pathways including skin development, keratinization, skin epidermis development and hair follicle development; Other cell clusters were also enriched in the corresponding development and differentiation related pathways (Fig. 4). These results further explained the rationality of clustering.

**Fig. 5:**
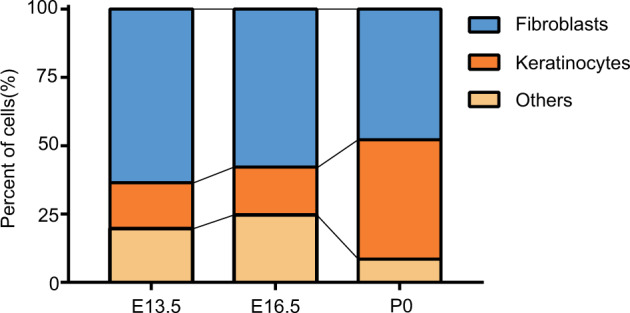
Percentage of specific cell type in E13.5, E16.5 and P0.

Additionally, the percentage of fibroblasts, keratinocytes and other cell types were counted (Fig. 5). It was found that the percentage of fibroblasts was gradually decreased (E13.5: 63.5%; E16.5: 57.8%; P0: 47.8%) and the percentage of keratinocytes was gradually increased (E13.5: 16.8%; E16.5: 17.5%; P0: 43.6%). This result was consistent with the development of hair follicles, dermal fibroblasts migrated directly to form DC structure^48,49^, dermal condensate cells as the precursors of dermal papilla/dermal sheath niche cells within the mature follicle^50^. Progenitor cell migrated and then formed the physically identifiable Pc^51^, placode cells as the earliest progenitors of all epithelial hair follicle cells including adult stem cells (SCs) were in the bulge^52^. Pc progenitors signal backed to the dermis for the formation of DC^6,53^. The formation of Pc and DC was the beginning of hair follicle development^54^. Then, DC structure formed dermal papilla (DP) through DC1 and DC2^55,56^, which enwrapped with the most-proximally located keratinocytes. Finally, keratinocyte proliferation led to the formation of hair germ, further down-growth progresses to the peg stage and the HF became morphologically noticeable^5^.

**Fig. 6:**
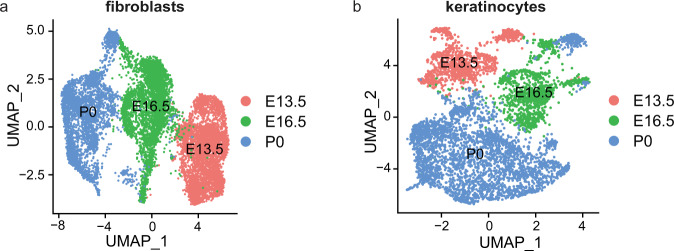
UMAP visualization of fibroblasts and keratinocytes integrated from E13.5, E16.5 and P0. (**a**) fibroblasts, (**b**) keratinocytes.

**Fig. 7:**
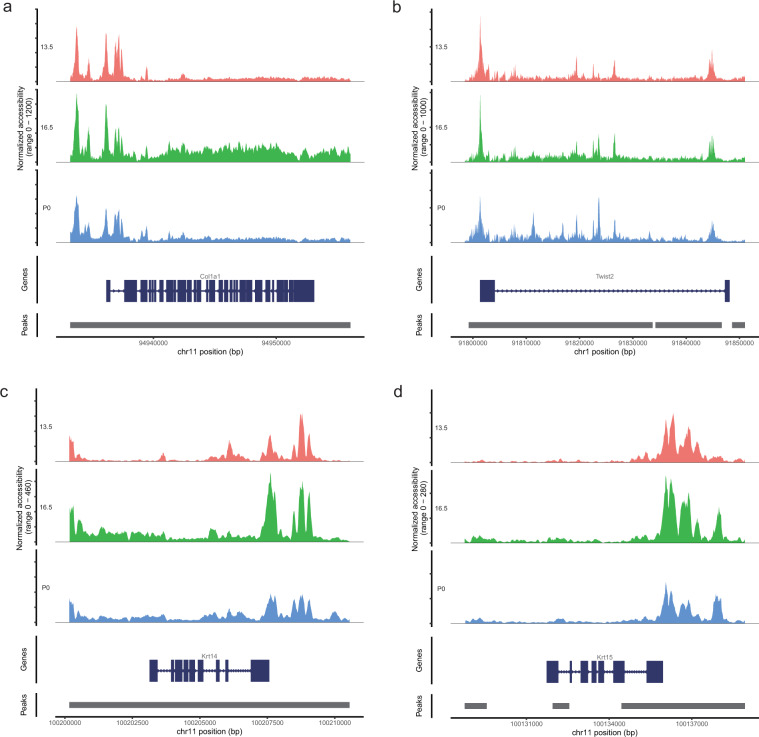
Chromatin accessibility of fibroblasts and keratinocytes markers at E13.5, E16.5 and P0. Chromatin accessibility of fibroblasts markers *Col1a1* (**a**) and *Twist2* (**b**) in fibroblasts. Chromatin accessibility of keratinocytes markers *Krt14* (**c**) and *Krt15* (**d**) in keratinocytes.

**Fig. 8:**
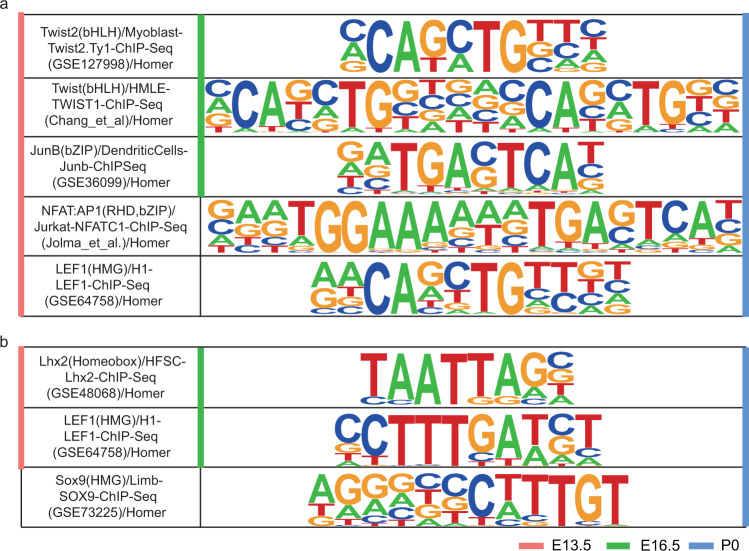
Motif Enrichment in differential peaks between E13.5, E16.5 and P0. (**a**) fibroblasts, (**b**) keratinocytes.

Finally, we integrated the fibroblasts and keratinocytes cluster in different developmental stages (Fig. 6), and performed further analysis. Chromatin accessibility analysis identified the differential accessibility regions (DARs) of fibroblasts (Supplementary Table 4, available at Figshare^26^) and keratinocytes (Supplementary Table 5, available at Figshare^26^) among E13.5, E16.5 and P0. Aligning to the reference genome, the DARs were annotated to promoter, intron, exon, 3′ UTR, etc. In the annotation information of fibroblasts (Supplementary Table 6, available at Figshare^26^) and keratinocytes (Supplementary Table S7, available at Figshare^26^), we focused on the regions which were annotated by the fibroblasts (*Col1a1* and *Twist2*) and keratinocytes (*Krt14* and *Krt15*) markers. We identified different DARs in the fibroblast cells and keratinocytes cells. The chr11-94912196-94914313 region was annotated to the distal intergenic region of *Col1a1* (Fig. 7a), and chr1-91737593–91738868, chr1-91848615-91866615 were annotated to the distal intergenic region of *Twist2* (Fig. 7b) in fibroblast cells. Meanwhile, the chr11-100218634-100219710, chr11-100221566-100222419, and chr11-100199847-100210887 were annotated to the distal intergenic region of *Krt14* (Fig. 7c), and chr11-100127801-100129561 were annotated to the UTR region of *Krt15* (Fig. 7d) in keratinocyte cells. It is generally believed that the accessibility of promoter regions is related to gene expression, the differential peaks in the promoter of *Krt14* and *Krt15* may play an important role in regulating gene expression. Meanwhile, we performed motif enrichment analysis on DARs at different stages of fibroblasts and keratinocytes, the enrichment results were shown in Supplementary Tables 8 and 9 (available at Figshare^26^). We found the stage differential peaks in fibroblasts were significantly enriched in *Twist2*, *Junb* and *Nfatc1* and *Lef1*, respectively (Fig. 8a). These transcription factors were related to the development of dermal fibroblasts^6^. The stage differential peaks of keratinocytes were significantly enriched in *Lhx2*, *Lef1* and *Sox9*, respectively (Fig. 8b). *Lhx2* is a transcription factor positioned downstream of signals necessary to specify hair follicle stem cells^57^. *Sox9* was an important transcription factor in dermal fibroblasts^6^. This result provided a basis for explaining reciprocal epithelial-mesenchymal signaling and was essential for the morphogenesis of mouse dorsal skin^58^.

**Fig. 9:**
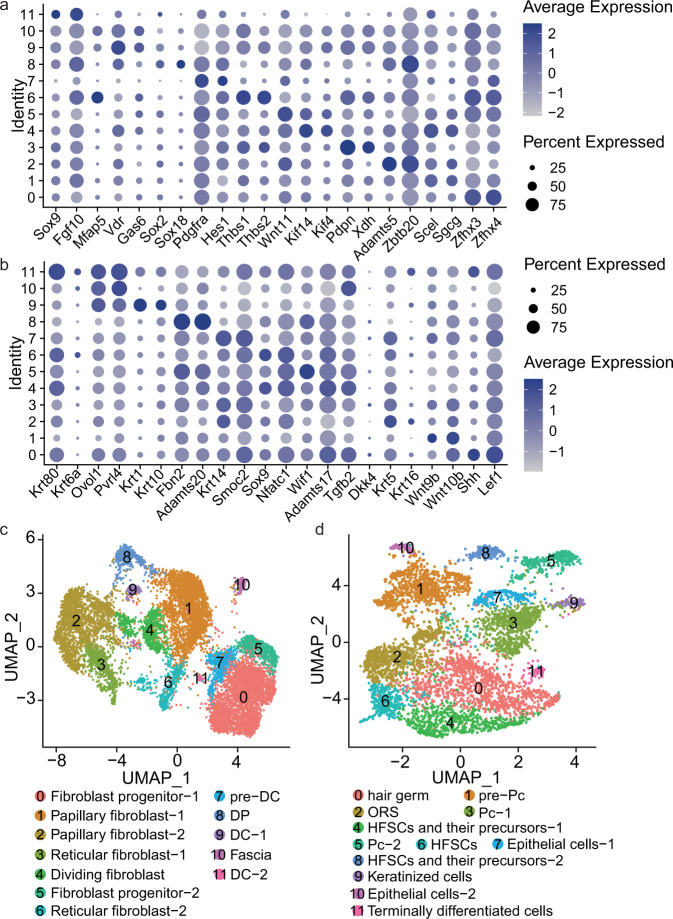
Subcluster fibroblasts and keratinocytes by gene activity. (**a**) Dot plots showing gene activity of marker genes for fibroblasts subtypes. (**b**) Dot plots showing the expression of marker genes for keratinocyte subtypes. (**c**) UMAP plots showing single-cell chromatin accessibility analyzed in fibroblasts. (**d**) UMAP plots showing single-cell chromatin accessibility analyzed in keratinocytes.

To better understand the heterogeneity of fibroblasts and keratinocytes clusters which were integrated from different developmental stages, we subclustered the fibroblasts and keratinocytes into 12 clusters, respectively. In fibroblast cells, we found that clusters 0 and 5 expressed fibroblast progenitor markers of *Zfhx4*, *Zfhx3* and *Wnt11*^6,59^, respectively, cluster 1 expressed papillary fibroblast markers of *Scel* and *Sgcg*^30^, clusters 2 expressed papillary fibroblast markers of *Adamts5* and *Zbtb20*^30^, cluster 3 expressed reticular fibroblast markers of *Pdpn* and *Xdh*^30^, cluster 4 expressed dividing fibroblast markers of *Kif4* and *Kif14*^30^, cluster 6 expressed reticular fibroblast markers of *Thbs1* and *Thbs2*^30^, cluster 7 expressed pre-DC markers of *Pdgfra* and *Hes1*^56^, cluster 8 expressed DP markers of *Sox18* and *Sox2*^6^, cluster 9 expressed DC markers of *Vdr* and *Gas6*^56^, cluster 10 expressed Fascia markers of *Mfap5*^30^, cluster 11 expressed DC markers of *Sox9* and *Fgf10*^56^ (Fig. 9a). In keratinocyte cells, we detected that clusters 0 expressed hair germ markers of *Shh* and *Lef1*^36^, cluster 1 expressed pre-Pc markers of *Wnt10b*^60^ and *Wnt9b*^36^, cluster 2 expressed outer root sheath (ORS) markers of *Krt5* and *Krt16*^36^, cluster 3 expressed Pc markers of *Dkk4*^36^, cluster 4 and 8 expressed hair follicle stem cells (HFSCs) and their precursor markers of *Tgfb2*, *Adamts17*, *Fbn2* and *Adamts20*^36^, cluster 5 expressed Pc markers of *Wif1*^36^, cluster 6 expressed HFSCs markers *Sox9* and *Nfatc1*^63^, cluster 7 and 10 expressed epithelial cell markers of *Krt14*, *Smoc2*, *Pvrl4* and *Ovol1*^6^, cluster 9 expressed keratinized cells markers of *Krt1* and *Krt10*^36^, cluster 11 expressed terminally differentiated cells markers of *Krt80* and *Ly6d*^36^ (Fig. 9b). According to the gene activity of marker genes, the fibroblast cells were classified into 8 populations, including fibroblast progenitor, papillary fibroblast, reticular fibroblast, DC, pre-DC, DP, Fascia and dividing fibroblast (Fig. 9c). The keratinocyte cells were classified into 9 populations, including Pc, hair germ, pre-Pc, ORS, HFSCs and their precursors, keratinized cells, epithelial cell and terminally differentiated cells (Fig. 9d). The differential gene activity of different fibroblasts and keratinocytes subtypes were shown in Supplementary Table 10 (available at Figshare^26^) and Supplementary Table 11 (available at Figshare^26^). The differential peaks of different fibroblast and keratinocyte subtypes were shown in Supplementary Table 12 (available at Figshare^26^) and Supplementary Table 13 (available at Figshare^26^). Many studies have found that promoter accessibility is positively correlated with gene expression, and the strongest correlation may be related to the function of housekeeping genes^61^. However, recent studies have found that there is a weak or no correlation between promoter accessibility and the transcription level of some genes^62^. The mechanism of this regulation process needs to be further revealed.

Taken together, our datasets provided a valuable resource for deeply exploring the epigenetic regulation mechanism of cell heterogeneity.

## Supplementary information

Supplementary Figures

## Data Availability

The R code used for the analysis of scATAC-seq data is available on GitHub (https://github.com/fangli0909/scATAC-seq_sa).

## References

[1] Gupta K (2019). Single-cell analysis reveals a hair follicle dermal niche molecular differentiation trajectory that begins prior to morphogenesis. Dev Cell..

[2] Duverger O, Morasso MI (2009). Epidermal patterning and induction of different hair types during mouse embryonic development. Birth Defects Res C Embryo Today..

[3] Paus R, Müller-Rver S, Veen C, Maurer M, Handjiski B (1999). A comprehensive guide for the recognition and classification of distinct stages of hair follicle morphogenesis. J. Invest Dermatol..

[4] Saxena N, Mok K, Rendl M (2019). An updated classification of hair follicle morphogenesis. Exp Dermatol..

[5] Schmidt-Ullrich R, Paus R (2005). Molecular principles of hair follicle induction and morphogenesis. Bioessays..

[6] Sennett R (2015). An integrated transcriptome atlas of embryonic hair follicle progenitors, their niche, and the developing skin. Dev Cell..

[7] Fang R, Preissl S, Li Y, Hou X, Ren B (2021). Comprehensive analysis of single cell ATAC-seq data with SnapATAC. Nat Commun..

[8] Consortium., T. E. P. (2012). An integrated encyclopedia of DNA elements in the human genome. Nature..

[9] Fiers M (2018). Mapping gene regulatory networks from single-cell omics data. Brief Funct Genomics..

[10] Törnqvist G, Sandberg A, Hägglund AC, Carlsson L (2010). Cyclic expression of Lhx2 regulates hair formation. Plos Genet..

[11] Wang S (2018). The inconsistent regulation of HOXC13 on different keratins and the regulation mechanism on HOXC13 in cashmere goat (Capra hircus). BMC Genomics..

[12] Fernandez-Guerrero M (2020). Mammalian-specific ectodermal enhancers control the expression of Hoxc genes in developing nails and hair follicles. Proc Natl Acad Sci USA.

[13] Ge W, Tan SJ, Wang SH, Li L, Wang X (2020). Single-cell transcriptome profiling reveals dermal and epithelial cell fate decisions during embryonic hair follicle development. Theranostics..

[14] Lin L, Zhang L (2022). Joint analysis of scATAC-seq datasets using epiConv. BMC Bioinformatics..

[15] Satpathy AT (2019). Massively parallel single-cell chromatin landscapes of human immune cell development and intratumoral T cell exhaustion. Nat Biotechnol..

[16] Buenrostro JD (2015). Single-cell chromatin accessibility reveals principles of regulatory variation. Nature..

[17] Chung CY, Ma Z, Dravis C, Preissl S, Wahl GM (2019). Single-cell chromatin accessibility analysis of mammary gland development reveals cell state transcriptional regulators and cellular lineage relationships. Cell Rep..

[18] Lyu P (2021). Gene regulatory networks controlling temporal patterning, neurogenesis, and cell fate specification in the mammalian retina. Cell Rep..

[19] Stuart T, Srivastava A, Madad S, Lareau CA, Satija R (2021). Single-cell chromatin state analysis with Signac. Nat methods..

[20] Stuart T, Butler A, Hoffman P, Hafemeister C, Satija R (2019). Comprehensive integration of single-cell data. Cell..

[21] Butler A, Hoffman P, Smibert P, Papalexi E, Satija R (2018). Integrating single-cell transcriptomic data across different conditions, technologies, and species. Nat Biotechnol..

[22] Fang L (2022). Gene Expression Omnibus.

[23] (2022). NCBI Sequence Read Archive.

[24] (2022). NCBI Sequence Read Archive.

[25] (2022). NCBI Sequence Read Archive.

[26] Fang L (2022). Figshare.

[27] Church RB, Schultz GA (1974). Differential gene activity in the pre- and postimplantation mammalian embryo. Curr Top Dev Biol..

[28] Joost S, Annusver K, Jacob T, Sun X, Kasper M (2020). The molecular anatomy of mouse skin during hair growth and rest. Cell Stem Cell..

[29] Driskell RR, Lichtenberger BM, Hoste E, Kai K, Watt FM (2013). Distinct fibroblast lineages determine dermal architecture in skin development and repair. Nature..

[30] Thompson SM, Quan MP, Winuthayanon S, Driskell IM, Driskell RR (2022). Parallel single cell multi-omics analysis of neonatal skin reveals transitional fibroblast states that restricts differentiation into distinct fates. J. Invest Dermatol..

[31] Gu LH, Coulombe PA (2007). Keratin function in skin epithelia: a broadening palette with surprising shades. Curr Opin Cell Biol..

[32] Ryncarz RE, Anasetti C (1998). Expression of CD86 on human marrow CD34(+) cells identifies immunocompetent committed precursors of macrophages and dendritic cells. Blood..

[33] Park J (2018). Single-cell transcriptomics of the mouse kidney reveals potential cellular targets of kidney disease. Science..

[34] Nonaka D, Chiriboga L, Rubin BP (2008). Sox10: a pan-schwannian and melanocytic marker. Am J Surg Pathol..

[35] Collins CA, Kretzschmar K, Watt FM (2011). Reprogramming adult dermis to a neonatal state through epidermal activation of β-catenin. Development..

[36] Morita R, Sanzen N, Sasaki H, Hayashi T, Fujiwara H (2021). Tracing the origin of hair follicle stem cells. Nature..

[37] Detmar M (1998). Increased microvascular density and enhanced leukocyte rolling and adhesion in the skin of VEGF transgenic mice. J. Invest Dermatol..

[38] Lim JH, Beg M, Ahmad K, Shaikh S, Choi I (2021). IgLON5 Regulates the Adhesion and Differentiation of Myoblasts. Cells..

[39] Fenger JM (2017). Abstract 3043: A novel Cpa3-Cre; miR-9 fl/fl mouse reveals a functional role for miR-9 in promoting mast cell invasion via up-regulation of CMA1. Cancer Res..

[40] Soler D (2006). CCR8 expression identifies CD4 memory T cells enriched for FOXP3+ regulatory and Th2 effector lymphocytes. J. Immunol..

[41] Beckers C, Simpson KR, Griffin KJ, Brown JM, Pease RJ (2017). Cre/lox Studies Identify Resident Macrophages as the Major Source of Circulating Coagulation Factor XIII-A. Arterioscler Thromb Vasc Biol..

[42] Blum R, Dynlacht BD (2013). The role of MyoD1 and histone modifications in the activation of muscle enhancers. Epigenetics..

[43] Zhang W (2018). Transcription factor EGR1 promotes differentiation of bovine skeletal muscle satellite cells by regulating MyoG gene expression. J. Cell Physiol..

[44] Belote RL (2021). Human melanocyte development and melanoma dedifferentiation at single-cell resolution. Nat Cell Biol..

[45] Feng L, Hwe C, Chu R, Meyskens F (2010). Abstract 2552: Loss of MiTF sensitizes melanoma cells to chemotherapy due in part to reduced LAMP1 accumulation. Cancer Res..

[46] Colombo S, Champeval D, Rambow F, Larue L (2012). Transcriptomic analysis of mouse embryonic skin cells reveals previously unreported genes expressed in melanoblasts. J. Invest Dermatol..

[47] Cho H, Kozasa T, Bondjers C, Betsholtz C, Kehrl JH (2003). Pericyte-specific expression of Rgs5: implications for PDGF and EDG receptor signaling during vascular maturation. FASEB J..

[48] Biggs LC (2018). Hair follicle dermal condensation forms via FGF20 primed cell cycle exit, cell motility, and aggregation. Elife..

[49] Glover JD (2017). Hierarchical patterning modes orchestrate hair follicle morphogenesis. PLoS Biol..

[50] Grisanti L (2013). Tbx18 targets dermal condensates for labeling, isolation, and gene ablation during embryonic hair follicle formation. J. Invest Dermatol..

[51] Ahtiainen L (2014). Directional cell migration, but not proliferation, drives hair placode morphogenesis. Dev Cell..

[52] Levy V, Lindon C, Harfe BD, Morgan BA (2005). Distinct stem cell populations regenerate the follicle and interfollicular epidermis. Dev Cell..

[53] Schindeler A (2008). Seminars in cell & developmental biology. Semin Cell Dev Biol..

[54] Chen D, Jarrell A, Guo C (2012). Lang R & R, A. Dermal β-catenin activity in response to epidermal Wnt ligands is required for fibroblast proliferation and hair follicle initiation. Development..

[55] Plikus MV (2017). Regeneration of fat cells from myofibroblasts during wound healing. Science..

[56] Mok KW, Saxena N, Heitman N, Grisanti L, Rendl M (2019). Dermal condensate niche fate specification occurs prior to formation and is placode progenitor dependent. Dev Cell..

[57] Rhee H, Polak L, Fuchs E (2006). Lhx2 maintains stem cell character in hair follicles. Science..

[58] Hardy MH (1992). The secret life of the hair follicle. Trends Genet..

[59] Driskell RR (2013). Distinct fibroblast lineages determine dermal architecture in skin development and repair. Nature..

[60] Zhang Y (2009). Reciprocal requirements for EDA/EDAR/NF-kappaB and Wnt/beta-catenin signaling pathways in hair follicle induction. Dev Cell..

[61] Li C (2022). The landscape of accessible chromatin in quiescent cardiac fibroblasts and cardiac fibroblasts activated after myocardial infarction. Epigenetics..

[62] Li Z (2019). Identification of transcription factor binding sites using ATAC-seq. Genome Biol..

[63] Nguyen MB (2018). FGF signalling controls the specification of hair placode-derived SOX9 positive progenitors to Merkel cells. Nat Commun.

