# Supplementary material for: Single-cell chromatin landscapes of mouse skin development
Source: Sci Data. 2022 Dec 2;9:741. doi: 10.1038/s41597-022-01839-9 (PMC9718782; doi:10.1038/s41597-022-01839-9)
Supplement: Supplementary file 1 — Supplementary Figures [file 41597_2022_1839_MOESM1_ESM.pdf]

Supplementary Figure1

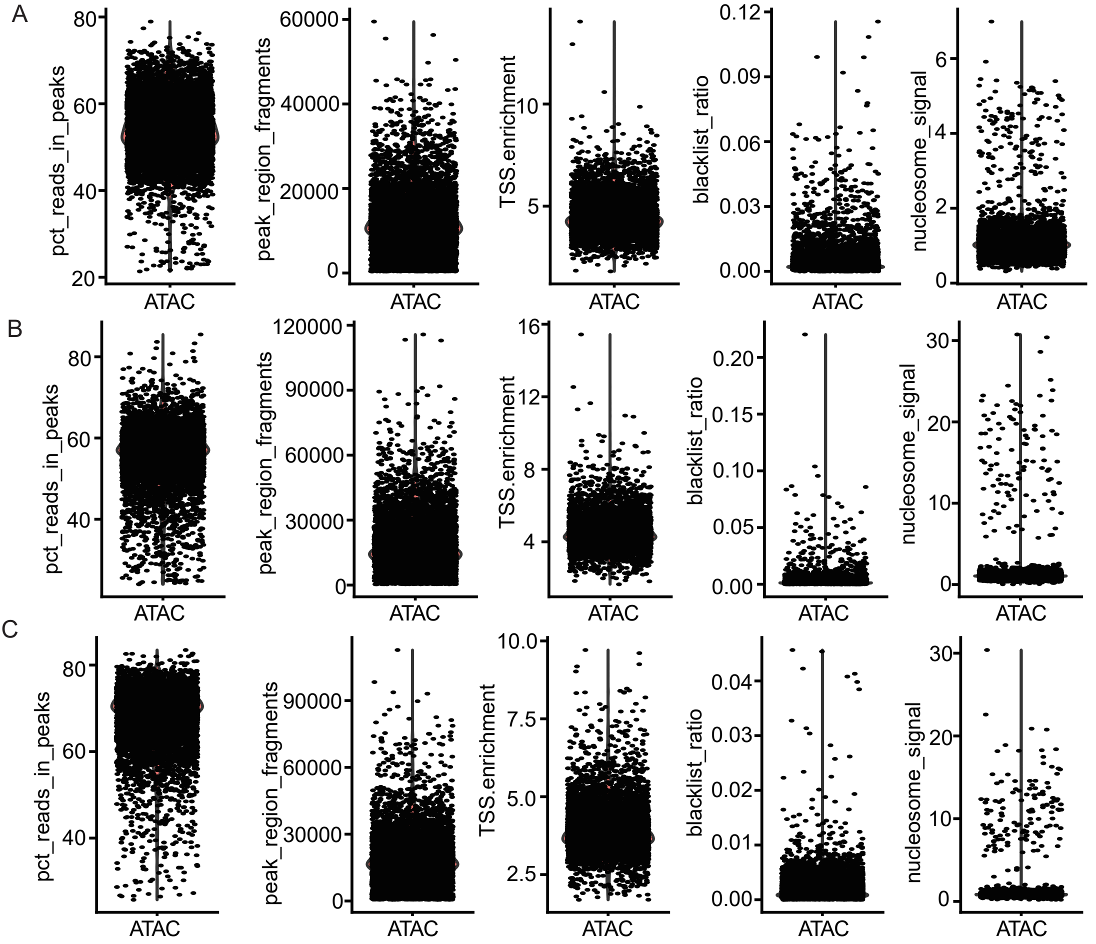

Supplementary Fig.1 Visualization of quality control index. (a) E13.5, (b) E16.5 and (c) P0

Supplementary Figure2

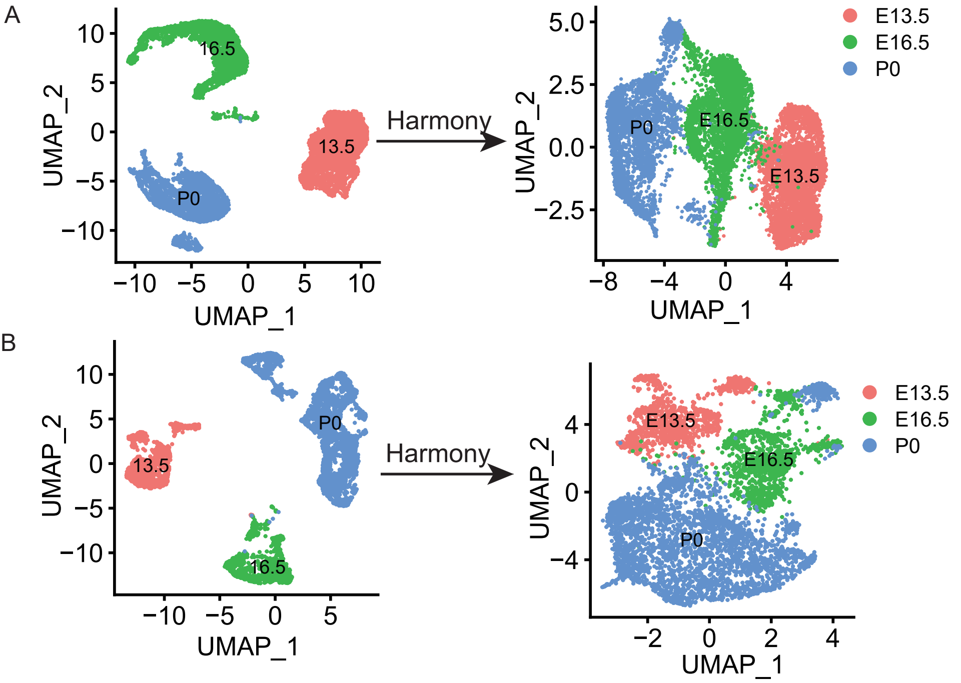

Supplementary Fig.2 Harmony scATAC-seq integration of fibroblasts(A) and keratinocytes(B).
